# Efficacy of thromboelastography in the management of anticoagulation for veno-venous extracorporeal membrane oxygenation in a coronavirus disease 2019 patient: A case report

**DOI:** 10.1097/MD.0000000000026313

**Published:** 2021-06-11

**Authors:** Ryosuke Nakashima, Masaaki Nishihara, Takeshi Iyonaga, Sho Iwasaka, Yuzo Yamamoto, Yuji Shono, Jun Maki, Kentaro Tokuda, Tomohiko Akahoshi, Taiki Higo, Takanari Kitazono, Hiroyuki Tsutsui

**Affiliations:** a Emergency and Critical Care Center, Kyushu University Hospital; b Department of Cardiovascular Medicine, Faculty of Medical Sciences, Kyushu University, Fukuoka, Japan.

**Keywords:** acute respiratory distress syndrome, anticoagulation, coronavirus disease 2019, extracorporeal membrane oxygenation, thromboelastography

## Abstract

**Rationale:**

In coronavirus disease 2019 (COVID-19) patients with acute respiratory distress syndrome refractory to optimal conventional management, we should consider the indication for veno-venous extracorporeal membrane oxygenation (V-V ECMO). Growing evidence indicates that COVID-19 frequently causes coagulopathy, presenting as hypercoagulation and incidental thrombosis. For these reasons, a multifactorial approach with several anticoagulant markers should be considered in the management of anticoagulation using heparin in COVID-19 patients on V-V ECMO.

**Patient concerns:**

A 48-year-old man was infected with COVID-19 with a worsening condition manifesting as acute respiratory distress syndrome.

**Diagnoses:**

He was refractory to conventional therapy, thus we decided to introduce V-V ECMO. We used heparin as an anticoagulant therapy for V-V ECMO and adjusted the doses of heparin by careful monitoring of the activated clotting time (ACT) and activated partial thromboplastin time (APTT) to avoid both hemorrhagic and thrombotic complications. We controlled the doses of heparin in the therapeutic ranges of ACT and APTT, but clinical hemorrhaging and profound elevation of coagulant marker became apparent.

**Interventions:**

Using thromboelastography (TEG; Haemonetics) in addition to ACT and APTT, we were able to clearly detect not only sufficient coagulability of COVID19 on V-V ECMO (citrated rapid thromboelastography-R 0.5 min, angle 75.5°, MA 64.0 mm, citrated functional fibrinogen-MA 20.7 mm) but also an excessive effect of heparin (citrated kaolin -R 42.7 min, citrated kaolin with heparinase 11.7 min).

**Outcomes:**

Given the TEG findings indicating an excessive heparin effect, the early withdrawal of ECMO was considered. After an evaluation of the patient's respiratory capacity, withdrawal from V-V ECMO was achieved and then anticoagulation was stopped. The hemorrhagic complications and elevated thrombotic marker levels dramatically decreased.

**Lessons:**

TEG monitoring might be a useful option for managing anticoagulation in COVID-19 patients on V-V ECMO frequently showing a hypercoagulative state and requiring massive doses of heparin, to reduce both hemorrhagic and thrombotic complications.

## 1 Introduction

Coronavirus disease 2019 (COVID-19) caused by severe acute respiratory syndrome coronavirus 2 leads to acute respiratory distress syndrome (ARDS),^[1]^ which sometimes requires veno-venous extracorporeal membrane oxygenation (V-V ECMO) as the only remaining treatment option.

Anticoagulation using heparin is recommended for patients on V-V ECMO,^[2]^ where it promotes thrombosis and a hypercoagulable state, concurrently with an increased risk of bleeding.^[3]^ It should be noted that coagulopathies of diverse etiologies have been frequently described in severe COVID-19 patients.^[4,5]^ For these reasons, there needs to be strict monitoring of the coagulation function during treatment with ECMO adjuvants. The activated clotting time (ACT) and activated partial thromboplastin time (APTT) are commonly used for monitoring on ECMO. However, a multifactorial approach is suggested due to the poor association between the heparin dose and APTT during V-V ECMO.^[6,7]^

**Figure 1:**
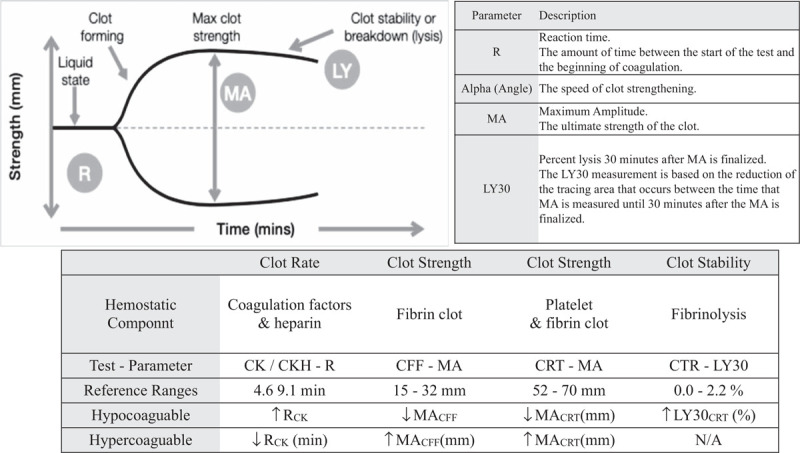
The TEG analyzer monitors the harmonic motion of a pendant drop of blood in response to external vibration. As the sample transitions from a liquid state to a gel-like state during clotting, the modulus of elasticity and resonant frequency increase. The analyzer measures these variations in resonant frequency during clotting and lysis. The greatest sensitivity to clotting factors and heparin is achieved with the R (reaction time) parameter of the CK and CKH tests. The clot strength is most rapidly assessed with the MA parameter of the CRT test, while CFF isolates fibrinogen contribution. CFF = citrated functional fibrinogen. CRT = citrated rapid thromboelastography.

Thromboelastography (TEG) is a fully established point-of-care viscoelastic test of hemostasis in whole blood that allows for the measurement of global clot formation and dissolution in real time (Fig. 1). It is largely used to guide transfusion in the perioperative management of cardiac surgery, liver transplantation, and trauma.^[8–10]^ Recently, Panigada et al. showed that TEG-guided anticoagulation in ARDS patients on V-V ECMO was associated with less heparin administration than a standard APTT-based protocol without increases in hemorrhagic or thrombotic complications, suggesting the potential utility of TEG in COVID-19 patients on V-V ECMO.^[11]^ To our knowledge, there have been no case reports describing effectiveness of TEG monitoring under anticoagulation in COVID-19 patients on V-V ECMO.

We herein report the excessive effect of heparin by TEG monitoring with no notable findings on an APTT evaluation.

## 2 Case presentation

Written informed consent was obtained from the patient for publication of this case report and any accompanying images. A copy of the written consent is available for review by the Editor-in-Chief of this journal. Because of this, there is no need to conduct special ethic review and the ethical approval is not necessary.

**Figure 2:**
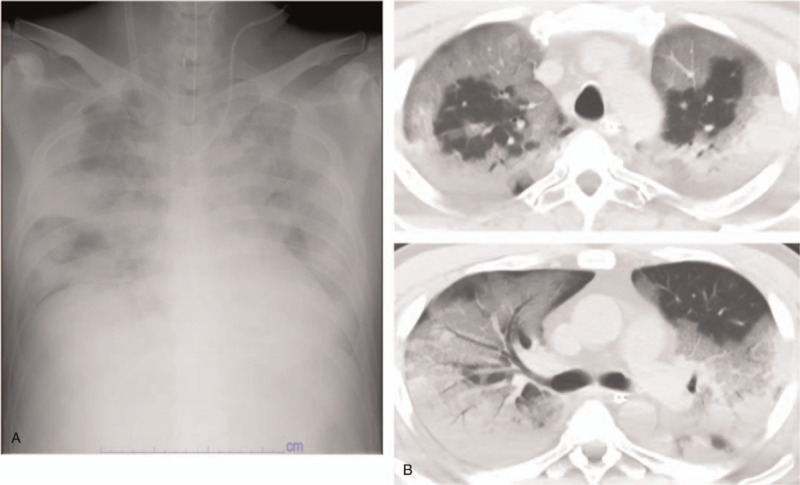
Left panel (A), bilateral alveolar infiltrates on chest x ray with normal cardiac size consistent with ARDS; right panel (B), a series of high resolution chest CT scans demonstrating bilateral extensive ground glass opacities. ARDS = acute respiratory distress syndrome.

A 48-year-old man (height; 175 cm, weight; 85.7 kg) had had symptoms of a fever, cough, and diarrhea since April 2 and was prescribed amoxicillin on April 6 at hospital A. Because of worsening symptoms of dyspnea, he was introduced to hospital B on April 9, and he was diagnosed with COVID-19 pneumonia. His past medical history included hypertension on medications and alcoholic liver disease, and active smoking. His hypoxemia progressively worsened, and he was finally intubated and placed on a ventilator on April 10. His respiratory status worsened, and the partial pressure of arterial oxygen/fraction of inspiratory oxygen ratio was < 100 on April 13, and then he was transferred to our hospital on April 14. Chest x ray and high resolution computed tomography scan on admission revealed bilateral diffuse alveolar infiltrates with normal cardiac size (Fig. 2[A]) and extensive ground glassing (Fig. 2[B]), respectively.

We tried to improve his oxygenation with ventilatory management combined with prone positioning, however, oxygenation was not improved. At that time, the partial pressure of arterial oxygen/FiO_2_ ratio was < 80 under ventilator support of fraction of inspiratory oxygen at 90% and positive end-expiratory pressure at 14 cmH_2_O, and his Murray's score was 3.25. Therefore, we decided to introduce V-V ECMO.

**Figure 3:**
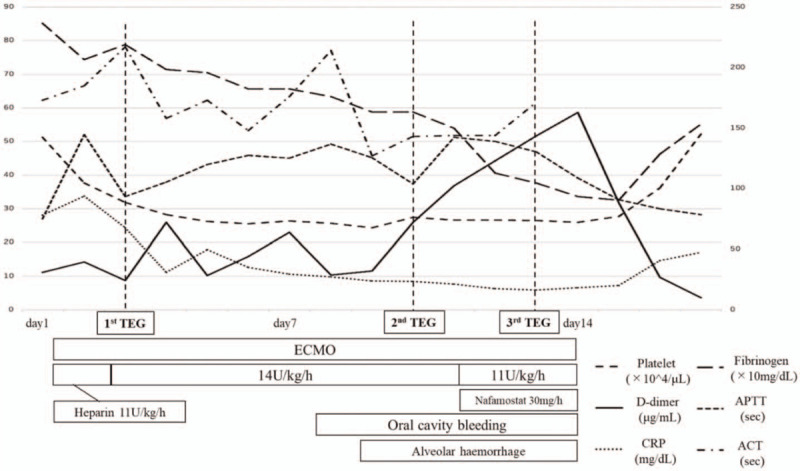
Clinical courses of post-hospitalization and timing of TEG examinations. ACT = activated clotting time, APTT = activated partial thromboplastin time, CRP = C-reactive protein, ECMO = extracorporeal membrane oxygenation, TEG = thromboelastography.

**Figure 4:**
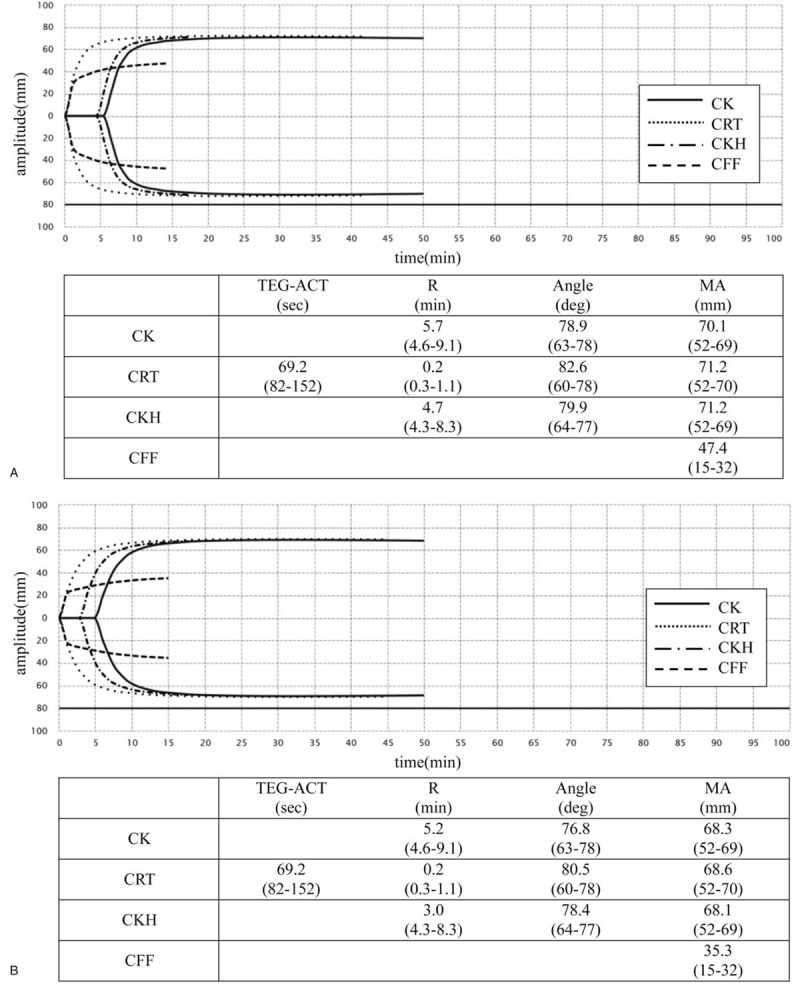
(A) TEG data of the patient evaluated on Day 3 after initiation of ECMO. 1st TEG shows the enhanced blood clot formation due to an increased function of fibrinogen. (B) TEG data of the patient evaluated on Day 10 after initiation of ECMO. 2nd TEG shows the sustained blood clot formation, probably due to the insufficient anticoagulation therapy.

Unfractionated heparin (11 U/kg) was initially administered for anticoagulation of V-V ECMO, and the dose was adjusted based on the ACT (therapeutic range: 140–180 sec) and APTT (therapeutic range: 40–65 sec). On Day 3 after the initiation of ECMO, the ACT and APTT were 217 and 33.7 sec, respectively, under 14 U/kg/h of unfractionated heparin. We also assessed TEG (1st TEG) on the same day, and the results showed that the blood clot strength was markedly enhanced (citrated rapid thromboelastography [CRT]-R 0.2 min, angle 82.6°, MA 71.2 mm, citrated functional fibrinogen (CFF)-MA 47.4 mm) due to an increased function of fibrinogen, while the effect of heparin was not sufficient (citrated kaolin [CK]-R 5.7 min, citrated kaolin with heparinase [CKH-R] 4.7 min) (Figs. 3 and 4[A]). The ACT was above targeted level and no clinical thrombotic event or increase in the D-dimer level was observed, thus we decided to continue the same dose of heparin.

On Day 10, the ACT (143 sec) and APTT (37.4 sec) had almost reached the therapeutic range under 14 U/kg/h infusion of heparin. However, TEG (2nd TEG) showed that the blood clot strength was still strong (CRT-R 0.2 min, angle 80.5°, MA 68.6 mm, CFF-MA 35.3 mm, CK-R 5.2 min, CKH-R 3.0 min), suggesting insufficient anticoagulation therapy (Figs. 3 and 4[B]).

**Figure 5:**
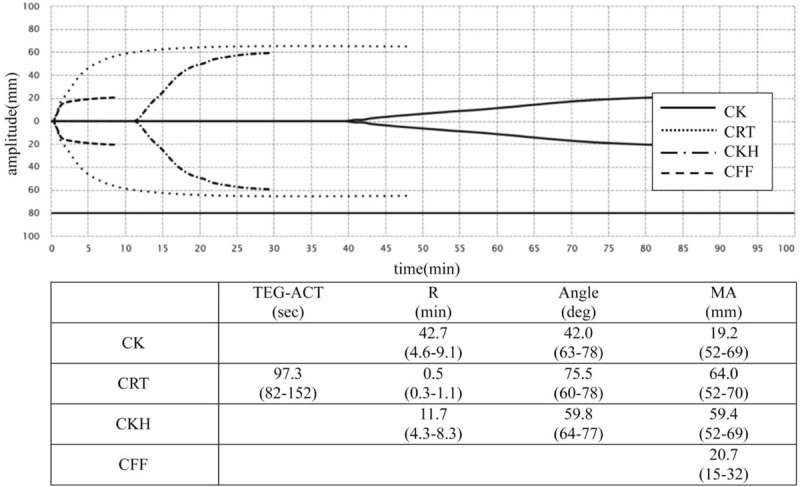
TEG data of the patient evaluated on Day 13 after initiation of ECMO. 3rd TEG revealed the excess effect of heparin that had not been shown by the ACT or APTT evaluation.

Hemorrhagic complications of oral bleeding and alveolar hemorrhaging became apparent around Day 8 after the introduction of the ECMO. We therefore reduced heparin (11 from 14 U/kg/h) on Day 10 and started continuous intravenous nafamostat mesilate (30 mg/h), which was relatively few bleeding complications. Under medication with these anticoagulants, the D-dimer/ fibrin/fibrinogen degradation products levels remained high, although the APTT and ACT were 46 and 140 seconds, respectively. Furthermore, there was no improvement in the oral, airway, or respiratory bleeding clinically. TEG was performed again on Day 13 (3^rd^ TEG) under 11 U/kg/h of heparin and 30 mg/h of nafamostat mesilate, clearly showing an excessing effect of heparin that had not been detected by the ACT/APTT evaluation along with a sustained strong blood clot function (CRT-R 0.5 min, angle 75.5°, MA 64.0 mm, CFF-MA 20.7 mm, CK-R 42.7 min, CKH-R 11.7 min; Fig. 5 in detail). These results might reflect the hypercoagulopathy associated with COVID-19 disease, in addition to ECMO treatment, despite the excessive effect of anticoagulation.

Given the 3^rd^ TEG findings indicating an excessive heparin effect, we considered the need for the early withdrawal of ECMO which was the best solution to thrombotic and hemorrhagic complications. On Day 14, withdrawal from V-V ECMO was achieved after an evaluation of the patient's respiratory capacity by several off-tests of VV-ECMO, and then anticoagulation was stopped. The hemorrhagic complications and elevated thrombotic marker levels dramatically decreased thereafter (Fig. 3). He was extubated on Day 16 and continued to receive oxygen along with rehabilitation. Fortunately, his respiratory condition gradually stabilize, and he was discharged from the intensive-care unit (ICU) on Day 28.

## 3 Discussion

In the present case, we found that TEG-guided anticoagulation therapy was useful for managing anticoagulation with heparin for a COVID-19 patient on V-V ECMO. In addition to conventional ACT and APTT, TEG revealed the excessive effect of heparin as the underlying cause of hemorrhagic complications. Given the hypercoagulative state and risk of hemorrhaging under anticoagulation using high doses of heparin in COVID-19 patients on V-V ECMO, TEG might play a crucial role in avoiding both critical complications.

Several coagulant tests, such as the APTT, ACT, prothrombin time (PT), fibrinogen concentration, and platelet count, are utilized in many clinical settings; however, we often face difficulties understanding the discrepancy between clinical bleeding or thrombosis and the results of these tests. TEG is an established way of measuring clot viscoelasticity using the whole blood and is used to predict coagulopathy and the risk of bleeding in surgery and anesthesiology.^[8,9]^ Accumulating evidence has demonstrated that TEG is useful for guiding anticoagulation therapy safely.^[8–10]^

TEG on Day 13 in the present case revealed an excessive anticoagulant effect of heparin (CK-R 42.7 min, CKH-R 11.7 min) although ACT and APTT were controlled within the optimal respective ranges during anticoagulant therapy with heparin. TEG makes it possible to assess the underlying coagulation with heparin by performing paired tests with and without heparinase, CKH and CK, respectively. Previous reports have shown that the TEG-guided anticoagulant therapy with heparin allowed the administration of lower heparin doses without an increase in either thrombotic or bleeding complications compared to the APTT protocol in patients with ECMO adjuvants,^[11]^ hemodialysis,^[12]^ and deep vein thrombosis.^[13]^ The results of the TEG examinations, including CK-R/CKH-R in the present case, provided supportive evidence for the reduction or discontinuation of heparin in anticoagulation therapy for this patient on ECMO when conventional APTT and ACT evaluations failed to provide useful data. TEG might be useful for managing anticoagulation in COVID-19 pneumonia patients in an excessive hypercoagulable state on V-V ECMO.

COVID-19 infection is reported to likely be associated with coagulopathy.^[14]^ The incidence of thrombotic complications is higher in COVID-19 patients than in other ICU patients,^[15]^ and an elevated D-dimer is an independent predictor for mortality.^[16]^ In the present case, TEG showed that the patient had a hypercoagulable status, supporting the above findings. TEG (Fig. 4[A] performed on Days 3) showed a marked increase in blood clot strength (CRT-R 0.2 min, angle 82.6°, MA 71.2 mm, and CFF-MA 47.4 mm). Furthermore, these results might suggest that the potentiation of fibrinogen played a crucial role in the hypercoagulation of this COVID-19 patient, as suggested by previous reports.^[14]^

## 4 Conclusions

We herein report a case in which TEG-guided anticoagulation therapy was useful in a COVID-19 patient with severe ARDS on V-V ECMO. In the future management of anticoagulation therapy in such cases, TEG evaluations might reduce both hemorrhagic and thrombotic events.

## Acknowledgments

The authors would like to thank Taku Yokoyama for his cooperation in the introduction and withdrawal of V-V ECMO in the present case.

## Author contributions

RN, TI, and MN contributed to the initial conception of the study and drafted the manuscript. All authors have read and approved the final manuscript.

**Data curation:** Ryosuke Nakashima, Masaaki Nishihara, Takeshi Iyonaga.

**Investigation:** Ryosuke Nakashima, Masaaki Nishihara.

**Writing – original draft:** Ryosuke Nakashima, Masaaki Nishihara, Takeshi Iyonaga.

**Writing – review & editing:** Masaaki Nishihara, Sho Iwasaka, Yuzo Yamamoto, Yuji Shono, Jun Maki, Kentaro Tokuda, Tomohiko Akahoshi, Taiki Higo, Takanari Kitazono, Hiroyuki Tsutsui.
